# Implementing recommendations to optimise professional support in the medical workplace: A participatory approach

**DOI:** 10.1111/medu.70054

**Published:** 2025-10-14

**Authors:** E. Reynolds, H. Lloyd, J. Cleland, G. Wong, L. Withers, T. Price, T. Gale, N. Brennan

**Affiliations:** ^1^ Faculty of Health University of Plymouth Plymouth UK; ^2^ Lee Kong Chian School of Medicine Nanyang Technological University Singapore; ^3^ Nuffield Department of Primary Care, Health Sciences University of Oxford Oxford UK; ^4^ University of Plymouth Plymouth UK

## Abstract

**Introduction:**

The professional support (including remediation) of practising doctors has not been widely researched, and there have been no studies to date that have implemented evidence‐based recommendations about support and remediation in the medical workplace. Our goal was to bridge the gap between research and practice in respect of optimising the delivery of professional support programmes for doctors in their workplace.

**Methods:**

We used a participatory‐action research (PAR) approach to implement recommendations from a previous study, RESTORE 1, in five UK sites: two hospitals, two professional support units and a professional support body. Informed by observations and interviews, we conducted a series of workshops (12 in total, with 35 relevant stakeholders [doctors, professional support leads, coaches etc.]). These were recorded and transcribed for analysis. Analysis was deductive, using the Promoting Action on Research Implementation in Health Services (i‐PARIHs) framework, the core constructs of which are innovation, recipient, context and facilitation.

**Results:**

Important aspects of innovation related to the perspective, language and tone of the recommendations and the finding that recipients often valued other types of evidence rather than research. In terms of the recipients, sites' motivation for engagement in the study was crucial. We identified a variety of enabling/constraining contextual factors including resources, type and role of organisation and responsibilities of the professional support programme, as well as macro‐level changes. The extent to which participants adopted the recommendations (facilitation) was limited.

**Discussion:**

The successful implementation of research into practice is challenging. However, new learning, shifts in relationships and increased awareness are equally valuable outcomes. ‘On‐the‐ground’ change takes time, depending on trust, relationships, partnership working and understanding context. Unfortunately, this does not align well with research systems that privilege studies with measurable outcomes produced within a set timeframe. We call for more discussion in medical education about the process of implementing research findings into practice.

## Abstract 2

Implementing evidence into doctors' professional support is complex. Using participatory action research, this study found that trust, relationships, and context—not quick fixes—drive meaningful change. #MedEd #PhysicianSupport #ImplementationScience

## 1 INTRODUCTION

There has been a dramatic growth in the literature on how to identify and support individuals who are struggling within medical education and training.^1^, ^2^, ^3^, ^4^, ^5^, ^6^ This large body of research has been distilled to provide guidance on best practices, practices to avoid and areas requiring further research.^7^, ^8^, ^9^, ^10^

Most of this literature focuses on those who are still considered ‘learners’ within the medical education and training system, particularly medical students.^2^, ^6^, ^8^, ^9^, ^11^ As such, the interventions or programmes proposed to support learners who are struggling are embedded into contexts characterised by clear learning objectives, competencies, milestones and performance assessments and faculty who are responsible for learners achieving the desired outcomes before being allowed to progress. However, no system is perfect, and once graduated, practising doctors can also experience performance issues at any stage in their careers and for many different reasons. The support mechanisms are less formalised, and the stakes of failure are arguably higher for those in training and consultants/attending physicians. However, these groups are relatively under‐researched, and supporting underperformance is an issue that employers and other organisations struggle to manage effectively.^1^, ^3^, ^10^, ^12^, ^13^

Before continuing, it is useful to define remediation and professional support. Professional support services exist to provide help, guidance and access to additional training and support for doctors in difficulty, to remedy problems and to help with career development.^14^, ^15^ Professional support is reactive, addressing particular needs or issues, and thus distinct from continuing professional development which is proactive approach to continuous learning.^16^ Remediation is a closely related concept which aims to facilitate the improvement of an individual whose competence has dropped below the expected level.^6^ Thus, professional support is broader incorporating the individual, the workplace, the team and the health care system.

In a previous study, RESTORE 1, we used a realist review approach to identify why, how, in what contexts, for whom and to what extent remediation programmes work for practising doctors.^10^, ^17^ This study developed recommendations to support the optimal tailoring, design and implementation of remediation interventions for underperforming doctors (those whose performance has fallen below the expected standards).^18^ The 14 recommendations are categorised into three main areas: developing insight, developing motivation and supporting behaviour change (see later).

However, these recommendations are merely the first step in a process of change: knowing what to do and doing are different things. Turning research evidence into policies and practices that work for people in the real world depends on knowledge translation, or implementation science, approaches to ‘promote the systematic uptake of research findings and other evidence‐based practices into routine practice … to improve the quality and effectiveness of a range of services’.^19^ Implementation science bridges the gap between research and practice, identifying and addressing barriers and facilitators to the uptake of evidence‐based innovations, by considering the ‘how’ (to implement effectively) as well as the ‘what’ (to implement). Without this, there is the danger of being stuck on a carousel of interventions that are not informed by research evidence, are not fit for purpose and do not support doctors effectively.

In this paper, we report on the process and outcomes of implementing the new knowledge gained in the RESTORE 1 project. Our goal was to bridge the gap between research and practice to optimise the delivery of professional support programmes for doctors in the UK workplace. To achieve this goal depended on stakeholder and researcher co‐engagement to leverage diverse expertise, co‐produce knowledge and solutions and create system‐level change for the integration of research evidence into practice.^20^, ^21^, ^22^ Thus, we adopted a participatory implementation approach, working with relevant stakeholders (professional support providers and their organisations) to consider how RESTORE 1 recommendations could be implemented into practice. As per Ramanadhan et al.^23^, ‘participatory approaches offer a range of benefits for implementation science, as stakeholders and researchers collaborate to influence the pathway from evidence to action’.

## 2 METHODS

### 2.1 Participatory research

Participatory action research (PAR) advocates for power to be shared, so researcher and researched are partners in the research process. The level of stakeholder engagement spans a wide spectrum, from community‐based participatory research (CBPR), which emphasises collaborative, equitable partnerships among researchers, stakeholders and community members throughout all phases of research; to collaboration, where stakeholders and researchers work together, but the researchers control decisions and resources; to consultation, where stakeholders are consulted for specific goals; and to the contractual, where stakeholders provide a site or setting for the research.^24^

The appropriate level of engagement relates to the goals and objectives for a given collaboration. Our goal and objectives were to use recommendations generated from a realist review of the literature^10^ to improve professional support services for doctors by developing action steps with the active involvement of those involved (doctors who have needed support and those who provide support services; see later for further details). Achieving these goals depended on stakeholders engaging and creating and ‘owning’ action steps to move research evidence into practice.^25^ With this in mind, our research aligns most closely with the collaboration approach defined above.^24^

### 2.2 Conceptual framework

Our approach to implementation aligned with the Stetler model^26^ and used the Integrated Promoting Action on Research Implementation in Health Services (i‐PARIHS) framework to organise data analysis (see later for further discussion). The i‐PARIHS framework positions successful implementation as the achievement of implementation goals, resulting from stakeholders facilitating an innovation in their (local, organisational and health system) context.^27^, ^28^, ^29^ The core constructs in the framework are innovation, recipient, context and facilitation (see Table 1).

**TABLE 1:** Characteristics of the innovation, recipients and context to be considered within the i‐PARIHS framework.^29^

Innovation	Recipients	Context
Focuses on sourcing and applying available research evidence to inform an innovation	Those who are both affected by, and influence (e.g. support or resist), implementation at an individual and collective team level	Micro‐, meso‐ and macro‐ levels of context that can act to enable or constrain implementation
Underlying knowledge sources Clarity Degree of fit with existing practices and values Usability Relative advantage Trialability Observable results	Motivation Values and beliefs Goals Skills and knowledge Time, resources, support Local opinion leaders Collaboration and teamwork Existing networks Power and authority Presence of boundaries	**Local level:** Formal and informal leadership support Culture Past experience of innovation and change Mechanisms for embedding change Evaluation and feedback processes Learning environment **Organisational level:** Organisational priorities Senior leadership and management support Culture Structure and systems History of innovation and change Absorptive capacity Learning networks **External health system level:** Policy drivers and priorities Incentives and mandates Regulatory frameworks Environmental (in)stability Inter‐organisational networks and relationships
**Facilitation**		
Facilitation is the active element in assessing, aligning and integrating the other three components (innovation, recipients and context)		

### 2.3 The research context

Context is a critical factor in determining which interventions are adopted, how they are adapted and what factors serve as barriers and facilitators to implementation.^23^, ^30^ Our context was a national‐level public health service where care is free at the point of contact, the United Kingdom's National Health Service (NHS). Our focal group was NHS hospital doctors, employed by local Trusts (semi‐autonomous organisational units within the NHS that manage one or more hospitals). There is wide variation in the support services provided for doctors across the United Kingdom in terms of how services are structured and the types of support available.^14^ See Supplementary File S1.

### 2.4 Sampling and recruitment

#### 2.4.1 Sites

**FIGURE 1:**
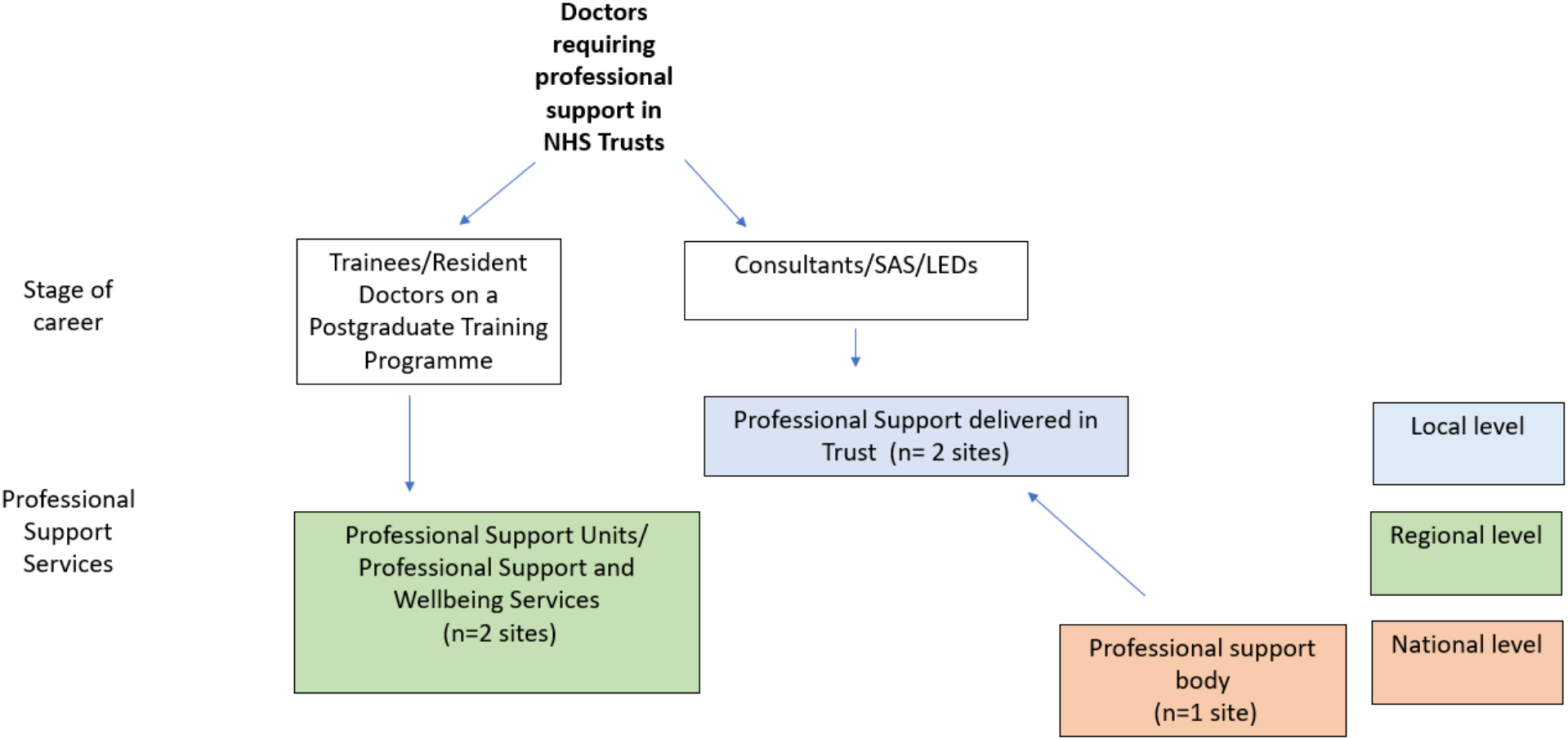
Stakeholder relationships and participant recruitment. [Color figure can be viewed at wileyonlinelibrary.com]

We purposively sampled and recruited five diverse sites willing to take part in this project. They included two NHS Trust hospitals (sites A and D), two NHS England (NHSE) Professional Support Units (PSUs) or Professional Support and Wellbeing Service (PSWS) (sites C and E) and a professional organisation (site B) (see Figure 1). Sites A, B and C had been involved in the RESTORE 1 stakeholder advisory group. At each site, a lead was recruited as the main point of contact for the study.

#### 2.4.2 Individuals

Site leads identified suitable professional support staff and doctors who had received professional support. Purposeful sampling was then used to recruit participants from both groups. We took care to invite staff with different roles in the professional support process to participate, to capture diverse perspectives.

Potential participants were invited to take part in the study via an email from the site lead. The invitation included a study information sheet, consent form, and details of whom to contact to discuss the project in more detail. Doctors who expressed an interest in taking part in the study were offered the choice of either joining workshops or being interviewed individually, in case those who had received professional support preferred a one‐to‐one interview due to the sensitivity of the topic. Data from the doctors' interviews were fed into the workshops anonymously (see below) to provide the doctor perspective and stimulate discussion.

### 2.5 Data collection

#### 2.5.1 Observations

We carried out observations^31^ at the sites to gain an understanding of the functioning of each professional support programme on the ground. The observed activities varied depending on what the site thought was appropriate. They included staff meetings, strategy meetings, and professional support staff outlining their role. The observations were recorded using Zoom or MS Teams where appropriate, or field notes were taken. Observations took place between November 2022 and May 2023.

#### 2.5.2 Individual interviews

The doctors interviewed individually were asked to tell us about their experience of professional support. They were presented with some examples of RESTORE 1 recommendations and asked to rank them in order of importance. They were asked about their views of potential outcome measures for professional support interventions. Where appropriate, relevant anonymous interview data were used in professional support staff workshops at different sites to stimulate discussion. Interviews were carried out remotely between October 2022 and September 2023 by phone (for some interviews), Zoom or MS Teams. Individual interviews lasted between 30 and 60 minutes.

#### 2.5.3 Workshops

We conducted a series of virtual workshops using MS Teams with each of the five sites. Two or three researchers (N.B., H.L. and E.R.) attended every workshop, with one researcher acting as chair and facilitating the discussion. Each workshop had a particular focus underpinned by the PAR principles of plan, act, observe and reflect, with a continuous circling back to reflection (Table 2).^32^, ^33^ In keeping with PAR, each workshop was participant‐led, and so they ran differently at each site. For example, some chose to discuss each recommendation in turn, and others chose to discuss the recommendations that were most important to their site. Workshops were carried out remotely between October 2022 and September 2023 by MS Teams. Workshops were held approximately 6–12 weeks apart to allow participants to reflect on the preceding workshop and take action on any of the changes discussed. Workshops lasted between 90 and 120 minutes.

**TABLE 2:** Workshop plans.

Workshop 1	Prior to the workshop, participants were sent the RESTORE 1 recommendations (Supplementary File S2: Table 5) and were asked to rank them in order of importance and state whether a recommendation was part of their service. In the shared version of the recommendations, the word ‘remediation’ was replaced with ‘professional support’ to encompass both remediation and professional support. At the beginning of the workshop, participants were reminded of the principles underpinning the nature and purpose of PAR workshops, i.e. equal power sharing, equitable partnership, and working together to problem‐solve. They were then asked to consider the usefulness of RESTORE 1 recommendations and discuss their rankings, and whether the recommendations could be implemented at their site to improve the service. Participants were also asked to give an overview of a typical professional support ‘journey’ of a doctor using their service, to identify any changes that could be made to improve the process, as well as potential outcome measures.
Workshop 2	Participants were asked to reflect on the first workshop. They were presented with the potential service developments they had identified, and were asked to discuss how their suggestions could be actioned.
Workshop 3:	Participants were presented with some initial findings from the RESTORE 2 research to date; for example, the differences in organisation and approaches to professional support between PSUs/PSWSs and Trusts, unequal access to support for different doctors, and the importance of early preventative measures. They were asked about their progress with the implementation of changes. Finally, they were asked for their reflections on the process of research and on their participation in the workshops.

### 2.6 Data management and analysis

All interviews and workshops were recorded using the Zoom/MS Teams recording function and/or a Dictaphone and were transcribed by a professional transcriber. Data were anonymised and stored securely on OneDrive for Business.

Data from the workshops and interviews was uploaded into Nvivo14 (QSR International) for analysis and coded deductively using the i‐PARIHS framework.^29^ One member of the research team (ER) carried out the preliminary coding and analysis. NB and ER met regularly to discuss and evolve the coding, which was also discussed in a larger research team meeting. The final stage of the analysis involved looking across the coded data to identify patterns, concepts, and themes.

### 2.7 Reflexivity and positionality

We used Guba and Lincoln's quality criteria to assess the rigour of our study.^34^, ^35^ In terms of credibility, we had a prolonged and varied engagement with the sites over an 11‐month period, and we ensured that researchers had the required knowledge and skills to perform their roles. Dependability was promoted by using a detailed research protocol throughout the study, as well as checking the accuracy of coding. Confirmability was addressed through discussion of the interpretation of data at regular meetings. Transferability was fostered by purposeful sampling of sites and participant types within sites to form nominated samples.

We built in regular opportunities for reflection and discussion in team meetings.^36^ To ensure diverse perspectives, the research team included multiple disciplines (e.g. psychology, clinical medicine and medical anthropology), a patient and public representative (PPI) and different professions (medicine, nursing and academic), specialties (anaesthesia and primary care) and gender. Interpersonal reflexivity in terms of considering the power dynamics in the workshops was particularly important for this study. Hence, the nature and purpose of PAR workshops were discussed with the research participants at the start of each workshop (see earlier). The researchers chairing the workshops had relatively little understanding of the delivery of professional support programmes in the NHS compared to the participants, which made the power dynamics more equitable. We reflected on our own positionality individually and in team meetings. Trust was built with participants through open discussions about ownership of information and their role in the process.

### 2.8 Ethics

This research study was reviewed and approved by the University of Plymouth Faculty of Health Research Ethics and Integrity Committee and the Health Research Authority Research Ethics Committee Wales 4 (IRAS#314766).

## 3 RESULTS

### 3.1 Overview

Eleven observations were held at sites B, C, D and E, and four interviews were conducted with doctors that had undertaken professional support at sites A, C and E (Table 3). Three workshops were held at sites C and E. Due to time constraints and participant availability, two workshops were held at sites A, B and D, combining the content of the second and the third workshops into one. There was an average of five participants in each of the 12 workshops (ranging from 2 to 10 per workshop).

**TABLE 3:** Overview of data collected for the study.

Site	No. of observations completed	No. of workshops	No. of professional support staff in all workshops	No. of doctors in all workshops	No. of doctors interviewed
A	0	2	4	2	1
B	2	2	11	0	0
C	4	3	7	0	1
D	1	2	4	0	0
E	4	3	9	0	2
Total	11	12	35	2	4

Interview and observation data were mostly used to gain an understanding of how the professional support programmes operated and informed the content of the workshops and are not reported separately.

A total of 35 professional support staff and two doctors took part in the 12 workshops. The professional support staff included coaches (n = 7), assessment and remediation managers (n = 8), human resources personnel (n = 3), administrators and any other relevant staff member involved in the professional support journey of a doctor, for example, advisors and associate deans (n = 12). Each workshop included the site lead and/or director of the professional support service (n = 5). Many of those working in some capacity in relation to professional support held multiple roles and took part in multiple workshops. Four doctors chose to take part in interviews instead of workshops.

Direct quotes from workshops are provided to support the results. These are identified by site and workshop; so, for example, C‐WKSP‐1 refers to Site C, first workshop. We have not specified individual workshop participants in quote labelling, to protect their anonymity.

Table 4 summarises the RESTORE 1 recommendations and the extent of their adoption across the sites prior to and during this phase of the study. None of the five sites adopted any of the RESTORE 1 recommendations within the timeframe of the study. However, some of the recommendations were existing or partially existing features of the programmes. The self‐directed format of the workshops (see Section 2) meant some topics were not discussed, and thus, a high level of ‘unknown data’ is reported in Table 4.

**TABLE 4:** Summary of adoption of RESTORE 1 recommendations at each site.^a^

	*Recommendations*	A	B	C	D	E
1.	**Remediation programmes work when they develop insight**.					
1.1	Doctors undergoing professional support should have the opportunity for confidential discussion with someone in a supportive role.	X	EF	EF	EF	EF
1.2	Professional support issues related to conduct should include an opportunity for doctors to reflect on their own professional values and contrast these with the feedback they receive on their own behaviours.	PEF	NR	EF	EF	EF
1.3	Doctors undergoing professional support should be supported by someone who has the role of advocate. This individual may be a coach or mentor, and should not have a role in making summative judgements throughout the professional support programme.	PEF	NR	EF	EF	EF
1.4	Doctors undergoing professional support should be provided with specific feedback that details the reasons and examples of underperformance or poor conduct. If the feedback relates to behaviour, it should detail specific events, with a date and time. This feedback should ideally come from more than one source and include feedback from patients whenever possible. Feedback will be needed throughout the professional support process, not just at the beginning. The appropriate feedback to determine progress, and the way that it is delivered, should be ascertained in the professional support planning stage.	EF	NR	NR	EF	NR
1.5	Feedback may be more effective when in person, and should be guided by someone who has been trained to deliver feedback. The feedback should be framed in such a way that it relates to the professional values of the doctor, is presented in a way that seems manageable, and affirms any identified strengths.	PE	NR	NR	UNK	NR
1.6	Multi‐modal assessment should be used to explore a full range of potential issues, including behavioural issues, even when the identified problem may appear to relate to knowledge and skills. Assessment should also be used to determine any organisational issues that may contribute to poor performance or behaviour. This will help determine whether the work environment is a contributory factor, and whether this environment will be suitable for undertaking professional support activities. If there are problems with the work environment, then professional support may need to be conducted elsewhere.	EF	EF	NR	EF	NR
1.7	Professional support programmes should offer the opportunity for the doctor to reflect on the reasons for their referral and to identify the triggers for under‐performance/poor conduct.	X	NR	PEF	EF	EF
2.	**Remediation programmes work when they motivate practitioners to change**.					
2.1	Where possible, doctors undergoing professional support should collaborate in the design of the individualised professional support plan and help to shape it. The planning stage should include setting scheduled points for assessing progress and determining what kind of feedback will be appropriate for the assessment of this progress.	UNK	EF	EF	UNK	EF
2.2	The doctor undergoing professional support should collaborate in the process of goal setting, and the goals set should be achievable and measurable.	UNK	EF	EF	UNK	EF
2.3	Professional support programmes should include an individualised plan that specifies the milestones, points for review of progress, and the consequences of achieving or not achieving targets.	UNK	EF	PEF	UNK	EF
2.4	Professional support programmes should seek to destigmatise the process of undergoing professional support and frame it, as far as possible, in terms of positive professional development.	UNK	UNK	EF	UNK	EF
3.	**Remediation programmes work when changes to practice are facilitated**.					
3.1	Where appropriate, professional support programmes should offer an opportunity for doctors undergoing professional support to practise any new skills or behaviours they have developed. This may include rehearsing new behaviours in simulated settings. Where this is not possible, guided reflection can offer an opportunity to reflect on in situ practice.	UNK	EF	EF	UNK	EF
3.2	Professional support programmes should have scheduled points for reviewing progress with the doctor. The doctor undergoing professional support should be involved in this process of review, and reflections should be guided so that the doctor continues to gain insight into their progress.	UNK	EF	NR	UNK	NR
3.3	Reflection should be built into the professional support programme and should be guided, but not form part of a final judgement on progress. Reflection may include one‐to‐one discussion of feedback, or discussions of entries in reflective logs. The purpose of reflection is to have an interesting and meaningful conversation to embed new knowledge and behaviours and engender further insight. Recent medico‐legal cases may have placed uncertainty over the confidentiality of reflective logs. The exact legal status of any written reflections should be established in advance.	UNK	EF	EF	EF	EF
		EF = 2	EF = 8	EF = 8	EF = 7	EF = 10
		PEF = 3	PEF = 0	PEF = 2	PEF = 0	PEF = 0
		✔ = 0	✔ = 0	✔ = 0	✔ = 0	✔ = 0
		X = 2	X = 0	X = 0	X = 0	X = 0
		NR = 0	NR = 5	NR = 4	NR = 0	NR = 4
		UNK = 7	UNK = 1	UNK = 1	UNK = 7	UNK = 0
		Total = 14	Total = 14	Total = 14	Total = 14	Total = 14

*Note*: EF = existing feature of programme. PEF = partially existing feature of programme. ✔ = adopted recommendation during timeframe of study. X = not an existing feature of the programme and did not adopt the recommendation during the timeframe of the study. NR = not relevant as not within the remit or role of the programme. UNK = unknown.

^a^ Data were drawn from tables completed by participants prior to workshop 1 plus qualitative data from the workshops.

The narrative results provide insight into what influenced participants' ownership and adoption of these recommendations. The results are organised by the four core constructs of the i‐PARIHS framework: innovation, recipient and context and facilitation.^29^

### 3.2 Innovation

Within the i‐PARIHS framework, ‘Innovation’ focuses on sourcing and applying available research evidence to inform an innovation.

Workshop discussions tended to focus on perceived barriers to applying RESTORE 1 recommendations in practice. The first barrier was that the recommendations focused on the individual as the locus of the ‘problem’, when the issues were often systemic.

There's a fundamental question here whether these kinds of recommendations reinforce a start which is deeply unhelpful, and actually what we would advocate is a much more systemic appreciative look at a kind of why someone is situated in a context whereby they are then labelled as being in difficulty. (C‐WKSP‐1)

There was some concern about the tone of the recommendations, particularly with the language used.

I was just struck by some of the language actually […] ‘issues related to conduct’ made me sort of sit back first of all […] that word is quite loaded and […] the connotations can be quite negative. (C‐WKSP‐1)

The word ‘support’ was seen to be ambiguous, making the recommendations unclear and difficult to implement.

I think one of the problems I have when reading this document is you're using ‘support’ to mean lots of different things … if you're looking at developing things that people can implement, it's almost separating out that pastoral support and developmental support because they are different. (D‐WKSP‐1)

The i‐PARIHS framework makes a clear distinction between research and evidence, with evidence being considered ‘broader’ than research, incorporating clinical experience, expertise and other sources such as audit information.^37^ It was clear that our participants valued other types of evidence to inform service change and development. It seemed that local knowledge and lived experience were valued more highly than the research‐based RESTORE 1 recommendations. For example:

A theme has emerged from advisors because of what the clients have fed back. […] that has [be]come a new pathway which we hadn't quite had before which is then supporting the supervisor and the trainee. (This has) totally emerged from the trainee's experiences and our advisors … it feels very responsive. (C‐WKSP‐1)

### 3.3 Recipients

Within the i‐PARIHS framework, recipients are those who are both affected by and influence (e.g. support or resist) implementation at an individual and collective team level.

Taking part in RESTORE 1 motivated three sites to take part in RESTORE 2. The Trust site D became involved because they wanted to publicise their proactive approach to improving professional support, and the second PSU site (E) became involved because they had an organisational goal of improving their professional support services.

However, motivation to take part did not equate to motivation to change. Where sites were confident that they provided a good professional support service, there seemed the attitude of ‘if it ain't broke, don't fix it’ irrespective of whether the RESTORE 1 guidelines may have informed further service improvement.

Interestingly, there was also scepticism of the research team's motivation and concern about what might happen to the data being collected.

I think initially when I saw this project I was a bit sceptical, I thought ‘well who wants to know and what's going to happen with the data and information’, because we were being scrutinised. (C‐WKSP‐3)

This may have affected the way participants engaged with the research project and recommendations. We discuss this in more depth later.

### 3.4 Context

In the i‐PARIHS framework, context includes micro‐, meso‐ and macro‐ levels of context that can act to enable or constrain implementation.

The nature of the organisation was relevant to implementing change. This was apparent in two ways. First, the professional support units (PSU) seemed to have greater freedom to change practice than Trusts, possibly because the focus of PSU work was support, whereas the focus of Trusts was patient care and health care delivery:

Most Trusts are focused very much on waiting lists and catch‐ups and various things like that, whereas we're not covered by any of that. We can just adapt ourselves to what our end users, whether they be our trainee groups or the wider stakeholder group and training programmes, and others, and patients ultimately […] need. (E‐WKSP‐3)

The second way in which the nature of the organisation was relevant was in respect of the ‘client group’ or remit of the site/organisation. Most doctors supported by a PSU/PSW (Sites C and E) were situated at the less serious end of the professional support spectrum whilst the professional organisation (Site B) usually operated at the more serious, regulatory end of the same spectrum. Trusts (Sites A and D) managed issues spanning the whole of this spectrum. However, the RESTORE 1 recommendations were most relevant to serious issues, and thus the PSUs did not see many of the recommendations as relevant to their service and consequently did not adopt them.

There are others [recommendations] that are very much along what I would call the sort of performance management approach that are rather reminiscent of what NCAS [National Clinical Assessment Service] used to do where they'd have an action plan and they'd look for insight, […] and, milestones of progress and how they'd be judged […] that really doesn't describe the kind of work that we are doing, […] You know, it really is very different to what we do. (C‐WKSP‐1)

Is our professional service to help people just get to point zero where they're coping or managing or is it about getting our staff to be thriving and flourishing? That's not coming across so much because of this kind of remedial aspect to it. (E‐WKSP‐1)

Conversely, Site B had implemented many of the RESTORE 1 recommendations prior to participating in the RESTORE 2 study because they aligned closely to their role and remit (i.e. the regulatory end of the spectrum).

I can't think of any major changes, because obviously the recommendations from the RESTORE 1 project were implemented quite closely in our process over the past sort of years. (B‐WKSP‐1)

A key contextual factor in a site's capacity to implement changes was access to resources, including time, finances, equipment and skills:

I think we've got all the bits to do it, you've got the mentoring […] but again this Trust doesn't resource it properly. It doesn't give you enough education [and] training and it doesn't give you the time to do it properly. (A‐WKSP‐1)

Finally, in relation to context, our sites did not exist in isolation. They were affected by wider organisational issues and change, from a national level restructuring of health and health education services in England during the time of data collection (April 2023), to new unit leadership.

So there's a lot going on, […] it's actually quite difficult to know […] to what extent being in the study has stimulated things, to what extent we would talk about it anyway, and to what extent these other pressures and drivers outside are involved. (C‐WKSP‐3)

### 3.5 Facilitation

Facilitation is the way in which the evidence was introduced or facilitated into practice.^29^ The i‐PARIHS framework attributes the success or otherwise of implementation to the ability of the facilitator and the facilitation process to enable recipients within their particular context to adopt and apply the innovation.

As indicated in Table 4, the uptake into practice of the RESTORE 1 recommendations during the timeframe of the study was non‐existent. Some of the reasons are discussed earlier in the results section, but we also must reflect on who were the facilitators in the process of change. While our aim was to empower participants to facilitate change, in practice, it seemed as if the participants did not want this role, sometimes handing the power to the researchers. Interpreting a lack of engagement or willingness to act, a researcher tried to steer one of the participants into action:

And D maybe you could spearhead bringing the coaches and the supervisors […] together to have a chat, seeing as that was your idea, perhaps. (E‐WKSP‐2)

Yet, while the workshop process did not seem to empower participants to change, it did seem to increase participants' capacity for future efforts by generating ‘power‐from‐within’.^38^ First, being part of the research study seemed to have provided a safe space for sites to affirm and validate positive aspects of their service and discuss how it could be improved. For example,

But there is, there is much more we can do as an organisation systematically […], to make it clear both to the individual who's in trouble but also to the people who're managing that, where the support is and, and make sure […] it's being accessed […] consistently. (A‐WKSP‐2)

Second, it seemed to be empowering in terms of having a clearer understanding of what to do when something needed escalating.

I've got a really better understanding as a non‐medical person of what's available should we have any concerns during coaching. So, I don't know whether that's a direct impact of the workshop or whether we just happened to be talking about that as well, but it certainly helped me to think about those things, and (I) also now have a clear understanding of what's expected of me as a coach. (E‐WKSP‐3)

Facilitation in the workshops, it seems, was more successful in developing a deeper understanding of, and a greater capacity for reflection on, the participants' organisation than it was in identifying specific strategies for change and the requisite task delegation for that change. This reminds us of the often unpredictable and generative nature of participatory action, where human actors decide the priorities within their capabilities and needs.

## 4 DISCUSSION

To the best of our knowledge, this is the first study that looks at how to integrate research findings into practice in the area of professional support and remediation of doctors. We used the i‐PARIHS framework and its constructs of innovation, recipient, context and facilitation to help us do so.

Our goal was to bridge the gap between research and practice in optimising the delivery of professional support programmes for hospital doctors in the UK workplace. Specifically, we focused on the process and outcomes of implementing the new knowledge gained in the RESTORE 1 project in workplace settings.

We found many of the RESTORE 1 recommendations were already an existing feature of the programmes, demonstrating congruence between evidence and practice mediated by context. However, none of the sites adopted the recommendations (facilitation) during the process of the RESTORE 2 intervention. Enabling/constraining contextual factors included resources, type and role of organisation and responsibilities of the professional support programme as well as system‐level changes.

Expanding on the above, it seemed that a narrow focus on ‘remediation’ was problematic. The RESTORE 1 recommendations were based on global academic literature,^10^ which differentiates between educational interventions aimed at improving practice or supporting wellbeing and remediation as mandated support defined in relation to patient safety.^1^, ^2^, ^39^ For some of the sites, particularly PSUs, this distinction was neither practical nor helpful. It was not practical because it did not speak to the context in which they work, where no such distinction existed between the services provided to those requiring support for their own professional needs and wellbeing and those requiring support to ensure patient safety. And it was not helpful because some of the corresponding language in the recommendations, which framed the ‘problem’ in terms of individual performance or underperformance, had understandably negative connotations and was counter to their own endeavours to create a supportive environment. The importance of a common language in implementation is not unique to the topic of professional support.^40^, ^41^

In terms of facilitation, participants/sites seemed to ‘pick and choose’, valuing or prioritising specific recommendations that aligned with the focus of their unit or their way of thinking and organisational culture, rejecting others that did not. This was irrespective of our intervention (RESTORE 2). Even where there was a lack of enthusiasm for the recommendations, our participatory research design provided a forum in which sites could discuss and scrutinise developments in their own processes and systems; in other words, the recommendations catalysed discussions. This demonstrates how PAR can empower participants to become agents of change, with signs of emerging agency or shifting relationships among stakeholders.

An interesting finding was that recipients did not value research evidence more highly than other forms of evidence such as that based on personal experience or observation. This process of blending external explicit evidence (the evidence‐based recommendation) with tacit practice‐based knowledge (the experiences and practices of everyday support staff) is considered an important way of enhancing the compatibility of a proposed change.^29^ This finding challenges our positionality as researchers as we often believe that research evidence is superior to ‘non scientific’/anecdotal evidence.

While this study successfully facilitated participant reflection and dialogue through the workshops, the process appears to have stopped short of enabling participant‐led action.^33^ Instead, the activities seem to have remained largely within the scope of researcher‐facilitated engagement. We believe that participant‐led action did not materialise due to the differing positions of researchers and participants, and the assumptions we made at the beginning of the study about it being possible for change to be facilitated within the time frame of the project, combined with the pressures in the system. We cannot know if any resultant changes will take place within those sites outside of the timeframes of the project but we did learn what was, and what was not already being done, and why.

### 4.1 Implications for research, policy and practice

We found that an important aspect of the process was in developing shared, appropriate language around professional support and remediation. This was not simply about the choice of vocabulary, but involved developing a shared understanding of the topic itself, establishing what distinctions were more academic than practical and appreciating the diversity of contexts in which these complex interventions operate.

A critical aspect of the PAR approach was the evolving role of the researchers within the inquiry process. Engaging deeply with participants and the complexities of the setting necessitated a continual negotiation of positionality, authority and collaboration. As the study progressed, the researchers shifted from initial roles as external facilitators to more embedded co‐learners, prompting reflexive engagement with their own assumptions, practices and learning. This transformation was not incidental but integral to the PAR process, aligning with the tradition of action research as both a personal and collective journey of change.^42^

### 4.2 Strengths and limitations

PAR is rarely used in medical education research (see previous studis^43^, ^44^ for recent exceptions), possibly because it is challenging to engage participants in implementation and action. Successfully engaging health and care settings in inherently time‐consuming research such as this is an achievement in a context where many services are struggling to maintain service provision, and staff feel under extreme pressure.^45^, ^46^, ^47^

Our study design did not allow us to capture longitudinal processes (e.g. whether the recommendations were adopted after the study or how things changed over time) or if the views expressed by stakeholders differed over time. It would be interesting to return to the research field to see if there was any longer term impact of participating in the workshops.

As with any framework or theoretical lens, an approach other than i‐PARIHS may have foregrounded different aspects of the data.^48^

Finally, our study is carried out in the context of one country, so we cannot assume our findings are generalisable to other contexts. However, the messages from the study are pertinent to all providers supporting doctors in need, and our use of the i‐PARIHS framework helps with conceptual generalisability.^49^

## 5 CONCLUSION

Our goal with the work presented in this paper was to bridge the gap between research and practice in optimising the delivery of professional support programmes for hospital doctors in the UK workplace. Importantly, and highly relevant for others whose focus is translating research into practice, we learned that implementation is not easy. As researchers, we like things to be finished, to be done, to move onto the next project. But trying to change things on the ground depends on relationships not just processes, a deep understanding of context, trust and partnership working. Unfortunately, this does not align well with research systems where studies and discrete programmes of work that are expected to have measurable outcomes within a set timeframe are the ones that are funded. We call for more discussion in the medical education literature about the process of translating or implementing research findings into practice on‐the‐ground.

## AUTHOR CONTRIBUTIONS

NB, TP, GW, TG, HL, JC, and LW conceptualised the study. ER, NB, and HL collected the data and ER, NB, and TP led the data analysis, with input from GW, TG, HL, LW, and JC. ER wrote the first draft of the manuscript. NB and JC led the revision process of all subsequent iterations, with ER, GW, HL, LW, TP, and TG providing critical contributions and refinements to the manuscript. All authors read and approved the final manuscript.

## CONFLICT OF INTEREST STATEMENT

None of the authors have a conflict of interest to disclose.

## Supporting information


**Supplementary File S1:** Structure of support services for different types of doctors in the UK.

**Supplementary File S2:** Table 5 Summary of adoption of RESTORE 1 recommendations at each site.

## Data Availability

Research data are not shared.
