# Supplementary material for: Implementing recommendations to optimise professional support in the medical workplace: A participatory approach
Source: Med Educ. 2025 Oct 14;60(2):167–78. doi: 10.1111/medu.70054 (PMC12805214; doi:10.1111/medu.70054)
Supplement: Supplementary file 1 — Supplementary File S1: Structure of support services for different types of doctors in the UK. [file MEDU-60-167-s001.docx]

**Supplementary File 1: Structure of support services for different types of doctors in the UK**

**Hospital, consultants and SAS doctors**

For hospital consultants, and SAS doctors (Specialist, Associate Specialist and Specialty Doctors, who have a permanent position as a middle-grade hospital doctor) professional support is typically provided by their Trust. They may also be referred to the Practitioner Performance Advice Service (PPAS) by their employer if it is felt that concerns are too complex or severe to be managed internally. The PPAS is a national NHS body offering advice and guidance to employers on providing support to doctors by developing action plans to help a doctor return to work following, e.g., intervention for performance concerns. The service may also undertake extensive clinical performance assessments to ascertain a doctor's support requirements when returning to work.

**Doctors in postgraduate training**

For doctors in postgraduate training (typically referred to as residents or [until recently] trainees in the UK), support is provided by either their employer, regional Professional Support Units (PSUs) or Professional Support and Wellbeing Services (PSWSs), organized by different organizations in each of the four UK countries. Nationwide organizations which provide professional support for all doctors include, but are not limited to, the Practitioner Health Programme, the British Medical Association, and charities such as Doctors in Distress ( <https://doctors-in-distress.org.uk/).>
