# Supplementary material for: Implementing recommendations to optimise professional support in the medical workplace: A participatory approach
Source: Med Educ. 2025 Oct 14;60(2):167–78. doi: 10.1111/medu.70054 (PMC12805214; doi:10.1111/medu.70054)
Supplement: Supplementary file 2 — Supplementary File S2: Table 5 Summary of adoption of RESTORE 1 recommendations at each site. [file MEDU-60-167-s002.docx]

**Table 5 Summary of adoption of RESTORE 1 recommendations at each site**

|  | ***Recommendations*** | **A** | **B** | **C** | **D** | **E** |
| --- | --- | --- | --- | --- | --- | --- |
| 1. | **Remediation programmes work when they develop insight.** |  |  |  |  |  |
| 1.1 | Remediating doctors should have the opportunity for confidential discussion with someone in a supportive role. | X | ✔ | ✔ | ✔ | ✔ |
| 1.2 | Remediation programmes for issues related to conduct should include an opportunity for remediating doctors to reflect on their own professional values and contrast these with the feedback they receive on their own behaviours. | X | NR | ✔ | ✔ | ✔ |
| 1.3 | Remediating doctors should be supported by someone who has the role of advocate. This individual may be a coach or mentor and should not have a role in making summative judgements throughout the remediation programme. | X | NR | ✔ | ✔ | ✔ |
| 1.4 | Remediating doctors should be provided with specific feedback that details the reasons and examples of underperformance or poor conduct. If the feedback relates to behaviour, it should detail specific events, with a date and time.  This feedback should ideally come from more than one source and include feedback from patients whenever possible.  Feedback will be needed throughout the remediation process, not just at the beginning. The appropriate feedback to determine progress, and the way that it is delivered, should be ascertained in the remediation planning stage. | ✔ | NR | NR | ✔ | NR |
| 1.5 | Feedback may be more effective when in person, and should be guided by someone who has been trained to deliver feedback. The feedback should be framed in such a way that it relates to the professional values of the doctor, is presented in a way that seems manageable, and affirms any identified strengths. | X | NR | NR | U | NR |
| 1.6 | Multi-modal assessment should be used to explore a full range of potential issues, including behavioural issues, even when the identified problem may appear to relate to knowledge and skills.  Assessment should also be used to determine any organisational issues that may contribute to poor performance or behaviour. This will help determine whether the work environment is a contributory factor, and whether this environment will be suitable for undertaking remediation activities. If there are problems with the work environment, then remediation may need to be conducted elsewhere. | ✔ | ✔ | ✔ | ✔ | ✔ |
| 1.7 | Remediation programmes should offer the opportunity for the remediating doctor to reflect on the reasons for their referral and to identify the triggers for under-performance✔poor conduct. | X | NR |  | ✔ |  |
| 2. | **Remediation programmes work when they motivate practitioners to change.** |  |  |  |  |  |
| 2.1 | Where possible, remediating doctors should collaborate in the design of the individualised remediation plan and help to shape it. The planning stage should include setting scheduled points for assessing progress and determining what kind of feedback will be appropriate for the assessment of this progress. | UNK | ✔ |  | UNK | ✔ |
| 2.2 | The remediating doctor should collaborate in the process of goal setting, and the goals set should be achievable and measurable. | UNK |  |  | UNK | ✔ |
| 2.3 | Remediation programmes should include an individualised plan that specifies the milestones, points for review of progress, and the consequences of achieving or not achieving targets. | UNK | ✔ |  | UNK |  |
| 2.4 | Remediation programmes should seek to destigmatise the process of undergoing remediation and frame it, as far as possible, in terms of positive professional development.  If relevant, remediation programmes could consider changing the name from *remediation* to *professional support* or similar. Positive framing may also include changing the language around the guidance for remediation, to include terms that indicate support and development. | UNK |  | ✔ | UNK | ✔ |
| 3. | **Remediation programmes work when changes to practice are facilitated.** |  |  |  |  |  |
| 3.1 | Where appropriate, remediation programmes should offer an opportunity for remediating doctors to practise any new skills or behaviours they have developed. This may include rehearsing new behaviours in simulated settings. Where this is not possible, guided reflection can offer an opportunity to reflect on in situ practice. | UNK | ✔ |  | UNK |  |
| 3.2 | Remediation programmes should have scheduled points for reviewing progress with the remediating doctor. The remediating doctor should be involved in this process of review and reflections should be guided so that the remediating doctors continues to gain insight into their progress. | UNK | ✔ |  | UNK |  |
| 3.3 | Reflection should be built into the remediation programme and should be guided, but not form part of a final judgement on progress. Reflection may include one-to-one discussion of feedback, or discussions of entries in reflective logs. The purpose of reflection is to have an interesting and meaningful conversation to embed new knowledge and behaviours and engender further insight. Recent medico-legal cases may have placed uncertainty over the confidentiality of reflective logs. The exact legal status of any written reflections should be established in advance. | UNK |  |  | ✔ |  |
|  |  | ✔ n=3  X=5 | ✔ n=6 NR =5 | ✔ n=5 | ✔n=7 | ✔n=7 |

Key

**✔ = long-standing feature of existing programme, adopted before study or adopted during study.**

**P = partially adopted**

**X = did not adopt**

**UNK = unknown**

NR **= not relevant as not their remit or role**
